# Spatiotemporal Dynamics of Activation in Motor and Language Areas Suggest a Compensatory Role of the Motor Cortex in Second Language Processing

**DOI:** 10.1162/nol_a_00093

**Published:** 2023-03-08

**Authors:** Lili Tian, Hongjun Chen, Pyry Petteri Heikkinen, Wenya Liu, Tiina Parviainen

**Affiliations:** Department of Psychology, University of Jyväskylä, Jyväskylä, Finland; Centre for Interdisciplinary Brain Research, University of Jyväskylä, Jyväskylä, Finland; School of Foreign Languages, Dalian University of Technology, Dalian, China; Language and Brain Research Centre, Sichuan International Studies University, Chongqing, China; Department of Psychology and Logopedics, Faculty of Medicine, University of Helsinki, Helsinki, Finland; Faculty of Educational Sciences, University of Helsinki, Helsinki, Finland; Faculty of Information Technology, University of Jyväskylä, Jyväskylä, Finland

**Keywords:** motor cortex involvement, magnetoencephalography, native language, second language, language proficiency, abstractness

## Abstract

The involvement of the motor cortex in language understanding has been intensively discussed in the framework of embodied cognition. Although some studies have provided evidence for the involvement of the motor cortex in different receptive language tasks, the role that it plays in language perception and understanding is still unclear. In the present study, we explored the degree of involvement of language and motor areas in a visually presented sentence comprehension task, modulated by language proficiency (L1: native language, L2: second language) and linguistic abstractness (literal, metaphorical, and abstract). Magnetoencephalography data were recorded from 26 late Chinese learners of English. A cluster-based permutation *F* test was performed on the amplitude of the source waveform for each motor and language region of interest (ROI). Results showed a significant effect of language proficiency in both language and motor ROIs, manifested as overall greater involvement of language ROIs (short insular gyri and planum polare of the superior temporal gyrus) in the L1 than the L2 during 300–500 ms, and overall greater involvement of motor ROI (central sulcus) in the L2 than the L1 during 600–800 ms. We interpreted the over-recruitment of the motor area in the L2 as a higher demand for cognitive resources to compensate for the inadequate engagement of the language network. In general, our results indicate a compensatory role of the motor cortex in L2 understanding.

## INTRODUCTION

The engagement of the motor cortex in language processing has been intensively discussed within the framework of embodied cognition. Based on the embodied view, language processing, specifically semantic processing (i.e., processing of meaning), involves not only classic language-related regions but also the motor system to simulate the perceptual meaning conveyed by words (Barsalou et al., 2008; Fischer & Zwaan, 2008; Gallese & Lakoff, 2005; Pulvermüller & Fadiga, 2010; Zwaan, 2014). The embodied view of semantic processing has been supported by neuroimaging and electrophysiological studies during the past decade, showing neural activations and oscillations in the motor cortex during meaning understanding (Fargier et al., 2012; Fernandino et al., 2013; Klepp et al., 2014, 2015; Mollo et al., 2016; Moreno et al., 2013). In addition, the action-sentence compatibility effect (Glenberg & Kaschak, 2002) has been taken as evidence for the involvement of the motor system in action-related semantic processing. Faster response was found when the direction of movement is congruent with the direction conveyed by the sentence (Glenberg et al., 2008; Kaschak & Borreggine, 2008; Santana & de Vega, 2011; Zwaan & Taylor, 2006). However, some recent studies failed to replicate any such motor compatibility effect (Greco, 2021; Morey et al., 2022; Papesh, 2015).

Clinical studies have provided more direct evidence for the involvement of the motor cortex in semantic processing by investigating patients with motor impairment (e.g., Parkinson’s disease, or PD; Buccino et al., 2018; Cardona et al. 2014; Desai et al., 2015; Fernandino et al., 2013; Kargieman et al., 2014; Kemmerer et al., 2012; Monaco et al., 2019). These studies showed that the motor-impaired participants had a selective difficulty in comprehending the action-related words (e.g., kick), manifested as a lower accuracy rate, longer response time, and an absence or attenuation of modulation of motor responses in patients with PD, compared with the healthy control group. The revealed association of impaired motor skills and deficits in understanding action-related meaning would support the embodied account of semantic processing. However, some other lesion studies failed to find the causal effect of motor cortex impairment on the processing of action-related meaning (Maieron et al., 2013; Papeo et al., 2010). These studies showing the dissociation of motor impairment and motoric semantic processing question the necessity of the motor cortex in language processing.

The emerging controversial findings have stirred up critiques and reflections on the embodied assumptions of language processing. As has been pointed out, the rapidly growing popularity of the embodied account is likely to result in the ignorance of other potential interpretations (see, e.g., Chatterjee, 2010; Hauk & Tschentscher, 2013; Mahon, 2015; Mahon & Hickok, 2016). Studies with the embodied hypothetical stance tended to interpret the data within the theoretical framework of embodiment with a prior hypothetical bias. For example, results showing motor activation in language tasks have been monotonically interpreted as the result of mental simulation of motor-related meaning, and therefore taken as an additional piece of evidence to confirm the embodied assumption. However, activation of the motor cortex may not necessarily be due to the mental simulation of motoric meaning. It can be ubiquitous in the language processing in general (Meteyard et al., 2012; Tian et al., 2020) or related to other aspects beyond strict linguistic processing (Maieron et al., 2013).

### Functional and Epiphenomenal Role

The emerging controversial findings impelled researchers to re-examine the role of the motor cortex in language processing and test whether activations in the motor cortex reflect the retrieval of lexical-semantic information (functional role) or arise as a byproduct of post-semantic motor imagery (epiphenomenal role). Some studies attempted to disentangle the functional and epiphenomenal role by scrutinizing the temporal information of motor activations (García et al., 2019; van Elk et al., 2010). In van Elk et al.’s (2010) study, an early activation of the motor area indexed by the mu rhythm event-related desynchronization (ERD) was found preceding semantic processing (around 400 ms after onset) and sustaining in parallel with semantic processing (around 700 ms after onset). Based on the early latency of motor activation, it was concluded that motor activation primarily reflected lexical-semantic retrieval and integration rather than post-lexical motor imagery.

Compared with neurotypical studies, lesion (pathological and virtual transient dysfunctions caused by repetitive transcranial magnetic stimulation [rTMS]) studies offered a more direct pathway for scrutinizing the causal role of the motor cortex, since researchers were able to detect the causality by manipulating stimulations over the motor cortex (Bocanegra et al., 2017; Desai et al., 2015; Fernandino et al., 2013; Pulvermüller et al., 2005; Reilly et al., 2019; Vukovic et al., 2017). In Vukovic et al.’s (2017) study, rTMS was employed over the left motor cortex within 200 ms of word onset to examine whether the stimulation would affect the processing of hand-related action words and abstract words in the lexical decision task—which requires very shallow lexical-semantic processing—and semantic judgment task—which requires explicit access to action-related meaning processing. The stimulation impaired the comprehension of the action words but facilitated that of the abstract words, compared with the performance in the lexical decision task. The interruptive effect of stimulation on lexical-semantic processing suggested a functional role of the motor cortex in semantic processing. Consistent results were also reported among studies concerning motor disorders, where associations were found between the impairment in action performance and the impairment in action-verb processing (Bocanegra et al., 2017; Desai et al., 2015; Fernandino et al., 2013).

Conversely, some studies reported dissociations between motor impairment and action semantic deficits (Maieron et al., 2013; Papeo et al., 2010). In Maieron et al.’s (2013) study, functional magnetic resonance imaging (fMRI) was employed to examine functional connectivity between the language network and primary motor cortex (M1) in an action-verb naming task. Participants were patients whose lesions involved (or spared) the M1 and healthy controls. It was found that lesions in the M1 did not degrade the performance of the action-verb naming task compared with the healthy controls. Results of the functional connectivity further revealed a lack of task-modulated connectivity between the M1 and language network in the action-verb naming task for both lesion and healthy groups. These findings indicated an accessory rather than functional role of the motor cortex in the processing of action words.

### Gradations of Motor Cortex Involvement

Instead of confirming or refuting the embodied hypothesis, some studies turned to explore the degree of motor cortex involvement, such as whether the motor cortex was differentially involved in different language settings. As highlighted by Chatterjee (2010) and Meteyard et al. (2012), the discussion of the graded nature of embodiment would shed light on the role that the motor system plays in semantic processing.

The gradation of motor cortex involvement has been mostly explored from the perspective of language proficiency (L1: native language; L2: second language) (Birba et al., 2020; De Grauwe et al., 2014; Monaco et al., 2021; Tian et al., 2020; Vukovic & Shtyrov, 2014; Zhang et al., 2020). By employing a passive reading task involving action-related words, Vukovic and Shtyrov (2014) found that the engagement of the motor cortex was greater for L1 than L2 for German-English speakers, indexed by a stronger ERD for the L1 than the L2 at around 8–12 Hz (mu rhythm). The stronger ERD for the L1 was interpreted as the result of a more integrated perception-action circuit for the L1 lexical-semantic representation. In contrast, in our earlier fMRI study (Tian et al., 2020), stronger activation of the motor cortex was found for the L2 than the L1, which was interpreted as the consequence of higher demand for cognitive resources as compensation for a less proficient language. Similarly, Monaco et al. (2021) also reported greater motor excitability for the L2 (English) than the L1 (French) in an action-related semantic judgment task, indexed by a higher motor evoked potentials for the L2 when the TMS was given 275 ms after word onset. However, the authors only claimed a different degree of motor cortex involvement between L1 and L2 semantic processing without further interpreting the implications underlying such differences. On the other hand, a similar degree of motor cortex activation has been reported (De Grauwe et al., 2014) between the L1 (Dutch native speakers) and the L2 (German advanced learners of Dutch) groups in performing a lexical decision task involving cognates and non-cognates with motor or non-motor-related meanings. The study therefore concluded that the lexical-semantic representation of the L2 was adequate to induce a similar degree of motor activation relative to the L1.

In addition to language proficiency, the gradation of motor cortex involvement has also been explored by manipulating the level of linguistic abstractness (e.g., literal/metaphorical/abstract language; Desai et al., 2013; Schaller et al., 2017; Tian et al., 2020). In Desai et al.’s (2013) study, four levels of linguistic abstractness were manipulated at sentence level, including literal action, metaphorical action, idiomatic action, and abstract verb. The blood oxygen level dependent signals of fMRI showed attenuated activation in the motor regions with the increase of linguistic abstractness (literal > metaphor > idiom > abstract). In our earlier study (Tian et al., 2020), we reported a similar decremental trend of motor activation with a hierarchically decreasing pattern of motor cortex activation from the literal to the abstract verb phrases.

### The Present Study

Previous studies have advanced our understanding of the motor system in semantic processing by exploring the gradations of motor cortex involvement in different linguistic circumstances. However, the discussed studies using fMRI, electroencephalogram (EEG), or TMS lacked either temporal or spatial accuracy in describing brain activation. Combining spatial and temporal resolution is crucial for the comprehensive understanding of how (and when) the motor cortex contributes to language understanding since timing and source dynamics of brain activation needs to be extracted simultaneously from language and motor areas. Majority of previous studies only focused on the motor regions of interest (ROIs), while ignoring the simultaneous neural activities of the language regions. In the present study, we employed magnetoencephalography (MEG) with millisecond temporal resolution and sub-centimeter spatial resolution to explore the temporal activation dynamics of motor and language areas in semantic processing. Specifically, we aim to investigate whether the degree of the engagement of motor and language areas is modulated by language proficiency (native language and second language) and linguistic abstractness (literal, metaphorical, and abstract).

## METHODS

### Participants

A total of 26 participants (8 male, 18 female) were recruited from the University of Jyväskylä, Finland. Participants were Chinese-English speakers, who started to learn English at the mean age of 9.77 (*SD* = 2.73) and had an average of 16.38 years’ (*SD* = 4.67) experience in learning English. Participants had the Lextale vocabulary test (www.lextale.com; Lemhöfer & Broersma, 2012) to measure their L2 vocabulary knowledge (mean ± *SD*: 74.18 ± 8.35). All participants were right-handed with normal or corrected-to-normal vision. None of the participants reported having any history of neurological disorder. Participants gave informed consent prior to participation. Participants were compensated for their participation in the experiment. The study was approved by the ethics committee of the University of Jyväskylä. Two participants were excluded from data analysis due to the low accuracy rate in behavioral performance (below 75%, mean = 93.04%, *SD* = 6%), resulting in 24 participants in the final analysis.

### Experiment Design

To examine the effect of language proficiency and linguistic abstractness on the degree of motor cortex involvement, L1 and L2 experiments were designed. Within each experiment, the factor of linguistic abstractness was manipulated with a gradual increase of abstractness from literal to metaphorical to abstract conditions. Each trial consisted of two verb phrases, with the second verb phrase either semantically congruent or incongruent with the first one. Participants were required to perform a semantic judgment task, where they needed to judge whether the second verb phrase shared the same meaning as the first phrase by pressing the response buttons.

### Stimuli

A total of 180 verb phrases (60 in each condition) were used in both L1 and L2 experiment. The literal and metaphorical phrases contained an action-related verb, either hand or arm related. The abstract phrase connoted the same meaning expressed by the metaphorical one (Table 1). Phrases in the L1 experiment were semantically equivalent to those in the L2 experiment, with few exceptions in the metaphorical condition, due to the lack of Chinese equivalents of some English metaphorical expressions. The verb phrases in both L1 and L2 experiments shared the same syntactic structure: verb + object. A frequency norming test and familiarity rating test were conducted to ensure that stimuli across conditions did not differ significantly in the aspects of word frequency and word familiarity (*p* > 0.01). Motor-relatedness of all stimuli was evaluated on a 5-point scale (1: *not related at all*; 5: *very related*): L1 experiment (literal: 4.60 ± 0.40; metaphorical: 2.78 ± 1.24; abstract: 2.12 ± 1.23) and the L2 experiment (literal: 4.39 ± 0.58; metaphorical: 2.64 ± 0.96; abstract: 2.11 ± 1.04). Only the first verb phrase, which is independent of task-related strategic manipulations, was used for further MEG analysis.

**Table 1.** An exemplar of stimuli in the L1 and L2 experiment

	L1 (Chinese)	L2 (English)
Literal	**抓住胳膊**–握住胳膊	**seize the arm** – hold the arm
	**抓住胳膊**–摔伤胳膊	**seize the arm** – hurt the arm
Metaphorical	**抓住机会**–把握机会	**seize the chance** – grab the chance
	**抓住机会**–错过机会	**seize the chance** – give up the chance
Abstract	**珍惜机会**–爱惜机会	**cherish the chance** – appreciate the chance
	**珍惜机会**–放弃机会	**cherish the chance** – abandon the chance

*Note*. The L1 and L2 stimuli are semantically equivalent.

### Experimental Procedure

**Figure 1.**
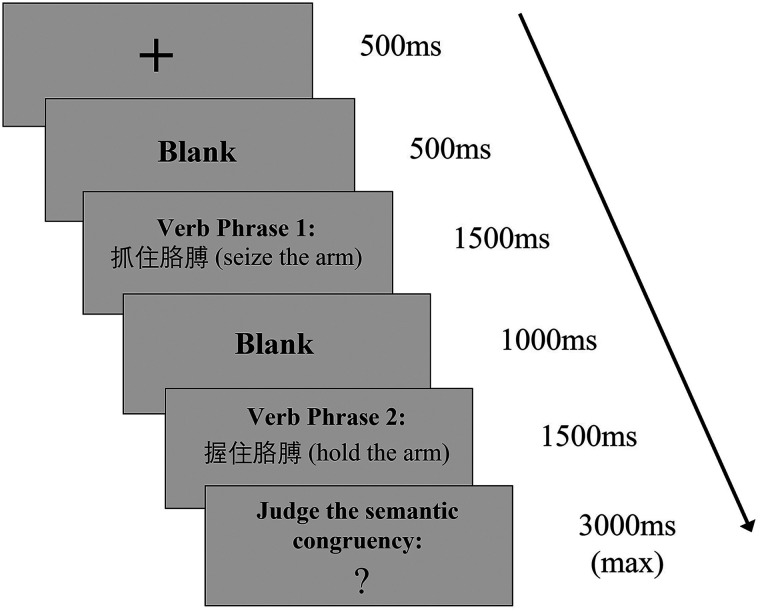
Schematic view of the experimental procedure.

L1 and L2 experiments shared the same experimental procedure. As suggested by previous studies, L1 could have a stronger translation priming effect on L2 than the other way around (i.e., asymmetrical cross-language priming effects; Chen et al., 2014; Keatley et al., 1994; Smith et al., 2019). To avoid the translation priming effect, L1 experiment was presented after L2 experiment. Trials were shown in a pseudo-randomized order. As shown in Figure 1, each trial began with a 500 ms fixation at the center of the screen, followed by a 500 ms long blank interval. Afterward, the first verb phrase was presented for a duration of 1,500 ms, followed by a 1,000 ms long blank interval. The second verb phrase was then presented for 1,500 ms, followed by “?” with a duration of maximal 3,000 ms. Participants were expected to give a response after the “?” appeared. Visual stimuli were presented using Presentation software (Neurobehavioral Systems, 2022). L1 stimuli were in KaiTi font and L2 stimuli in Times New Roman font. The viewing distance from participants’ eyes to the stimuli on the projection screen was one meter. The L1 stimuli subtended a horizontal visual angle of 3° 5′, and the L2 stimuli subtended a horizontal visual angle of 4° 58′.

### MEG Data Recording

Continuous neuromagnetic signals were recorded using a 306-channel (102 magnetometers and 204 planar gradiometers) whole-head MEG system (MEGIN Oy, 2022) in a magnetically shielded room at the Centre for Interdisciplinary Brain Research, University of Jyväskylä, Finland. The head position of each subject was monitored by five head-position indicator (HPI) coils attached over the forehead and behind each ear. Electrooculography signals were recorded simultaneously by four electrodes attached around the eyes: above/below the right eye, near the corner of the left/right eye. One ground electrode was attached to the collar bone. The position of three fiducial landmarks (nasion, left/right preauricular points), as well as approximately 120 digitization points over the scalp, were acquired to establish the head coordinate frame for the coregistration between MEG data and the MRI template. MEG signals were online bandpass filtered at 0.1–330 Hz with a sampling rate of 1000 Hz.

### MEG Data Preprocessing and Source Estimation

Raw MEG data were processed in MaxFilter 2.2 (Elekta, 2010) with the time-domain extension of the signal space separation method to minimize external magnetic disturbance and within-sensor artifacts and to compensate for head movement (Taulu & Kajola, 2005). Head position was estimated with a buffer length of 30 s and a correlation limit of 0.980. Head movement correction was performed using a 200 ms window with a 10 ms step. The error limit of HPI coil fit acceptance was 5 mm with a g-value of 0.98.

The preprocessing was performed with Meggie, a graphic user interface built in-house based on MNE-Python software (Gramfort et al., 2013). First, visual inspection was done to identify and exclude the bad data segments in the continuous MEG data. Then, MEG data were resampled to 250 Hz. A lowpass filter of 40 Hz (transition bandwidth 0.5 Hz, filter length 10 s) was applied. Physiological artifacts related to heartbeat, blink, and saccade were removed using a semiautomatic independent component analysis method. Event-related epochs were extracted from −200 ms to 1,000 ms relative to the onset of the first verb phrase. A 200 ms interval before the onset was used as the baseline. MEG epochs with an amplitude exceeding 3,000 fT/cm for gradiometers or 4,000 femtoteslas (fT) for magnetometers were rejected from further analysis.

In the calculation of evoked responses for the literal, metaphorical, and abstract conditions, the first verb phrase was combined across the congruent and incongruent trials. Evoked responses were obtained by averaging the signals of each condition (literal, metaphorical, and abstract) in each experiment (L1 and L2 experiment).

Source estimation was performed in MNE-Python (Version 0.17.0; Gramfort et al., 2013). The CN200 template (https://www.nitrc.org/projects/us200_cn200; Yang et al., 2020), based on T1-weighted magnetic resonance images of 250 healthy Chinese adults, was used for cortical reconstruction and volumetric segmentation. Coregistration between the CN200 template scalp and the digitized scalp was performed for each participant using a three-axis scaling mode. Shrunk covariance with cross-validation was used to estimate the noise-covariance matrix (Engemann & Gramfort, 2015).

Dynamic statistical parametric mapping (dSPM; Dale et al., 2000), which is based on minimum-norm estimate (Hämäläinen & Ilmoniemi, 1994), was used for source estimation with a source space consisting of 4,098 vertices and 4,098 loose-constraint and depth-weighted current dipoles (loose = 0.2, depth = 0.8) distributed on the cortical surface in each hemisphere. Source estimation results were then noise normalized using the dSPM. The source estimates across participants were morphed to the same cortical space (CN200 template).

### ROI Selection

**Figure 2.**
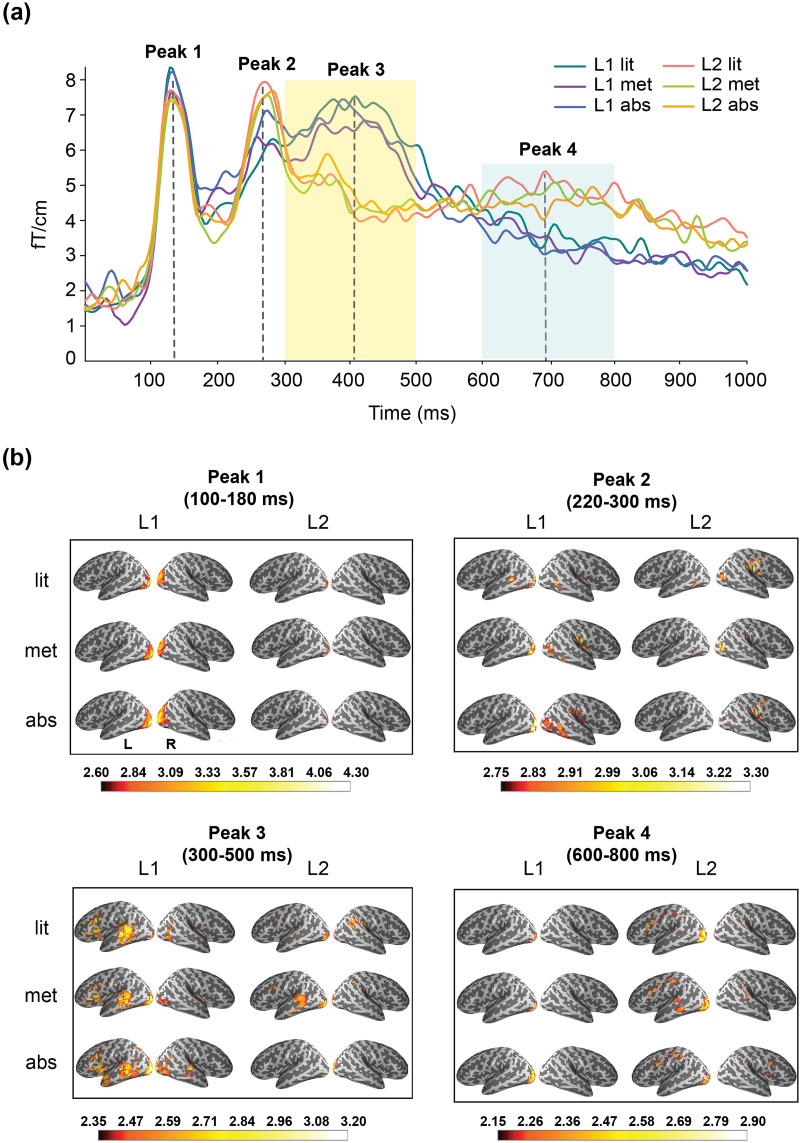
Grand-averaged results at sensor and source level. (A) Grand-averaged sensor waveform (204 gradiometers) across conditions in the L1 and L2 experiments; the light-shaded areas indicate the time window applied to the region-of-interest-based statistical analysis. (B) Grand-averaged source activation quantified as mean dynamic statistical parametric mapping (dSPM) value over time points corresponding to each peak: ±40 ms duration prior and after the relatively transient peaks (peak 1 and peak 2), and ±100 ms prior and after the relatively sustainable peaks (peak 3 and peak 4). The intensity of the color in the cortical activation map indicates the degree of dSPM value. L1: native language (Chinese); L2: second language (English); lit: literal; met: metaphorical; abs: abstract; L: left hemisphere; R: right hemisphere.

Regions of interest were selected in a hybrid way. First, based on the timing of peak activities in the grand-averaged sensor waveform (Figure 2A), the spatial distribution of cortical sources corresponding to each peak was identified (Figure 2B).

**Figure 3.**
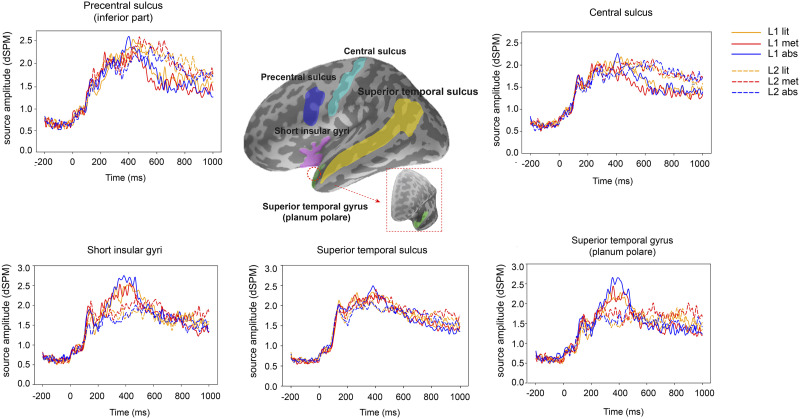
Grand-averaged source time courses for the literal, metaphorical, and abstract conditions in the L1 and L2 experiments in the indicated ROIs (language ROIs: short insular gyri, planum polare of the superior temporal gyrus, and superior temporal sulcus; motor ROIs: inferior part of the precentral sulcus and central sulcus). The parcellation of each ROI is shown in the inflated brain surface with a lateral view. For a better view, the planum polare of the superior temporal gyrus is also shown with a rostral view.

Next, the source distribution was compared against previous meta-analysis results of neuroimaging studies pertaining to semantic processing and motor performance/imagery. Brain regions appearing in both the data-derived cortical activation maps and previous meta-analyses were selected as ROIs for the present study. The selection was done by using MNE_analyze (https://mne.tools/0.17/manual/gui/analyze.html#the-labels-menu; Gramfort et al., 2014). First, label names corresponding to the literature-derived brain regions were selected from the parcellation list (Destrieux Atlas a2009s; Destrieux et al., 2010). Then, the partition of the selected region was overlaid with the MEG data on the inflated cortical surface. Only areas which showed prominent activation within the partitions were selected as ROIs. Both language and motor ROIs were selected left-lateralized due to only minor activation in the right hemisphere (Figure 2B). All ROIs were parcellated based on the Destrieux Atlas a2009s (Destrieux et al., 2010; see the schematic view of ROIs in Figure 3).

The above procedure resulted in the following language ROIs: short insular gyri (partially overlapping with inferior frontal gyrus; Binder et al., 2009; Friederici et al., 2003; Rueckl et al., 2015), planum polare of the superior temporal gyrus (part of anterior temporal cortex; Carreiras et al., 2013; Lambon Ralph et al., 2017; Patterson et al., 2007), and superior temporal sulcus (Citron et al., 2020; Rueckl et al., 2015). Motor ROIs were selected as the inferior part of precentral sulcus and central sulcus (part of primary motor cortex; Hari et al., 1998; Hétu et al., 2013; Michelon et al., 2006; Porro et al., 1996; Yousry et al., 1997). The ROI-based source time courses are shown in Figure 3.

### Time Window Selection

The time window was selected based on the latency of peak activities in the grand-averaged sensor waveform (Figure 2A) and the corresponding time-resolved source activation maps (Figure 2B). Based on the visual inspection, four peaks were identified in the sensor waveform: peak 1 at around 140 ms, peak 2 at 260 ms, peak 3 at 400 ms, and peak 4 at 700 ms.

Based on the source activation map, the first two peaks reflected activation in the visual cortex (peak 1) and more distributed areas across occipital-temporal lobes (peak 2), which were not included for statistical analysis. During peak 3 (300–500 ms, with peak activity at around 400 ms) and peak 4 (600–800 ms, with peak activity at around 700 ms), activation was found within temporal and frontal-central lobes, overlapping with our selected ROIs. Therefore, these two time windows, TW1 (300–500 ms) and TW2 (600–800 ms), were used for further statistical analysis.

### Statistical Analysis

Statistical analysis was performed on the amplitude of the source waveform (represented as dSPM value) extracted from each ROI separately for TW1 and TW2. To examine the effect of language proficiency and linguistic abstractness, the nonparametric two-way repeated measures analysis of variance with spatiotemporal clustering was performed in MNE-Python. To solve the multiple comparison problem, a cluster-based permutation test across time and space was employed (Maris & Oostenveld, 2007). The randomization times of the permutation test were 1,000, with a threshold for cluster inclusion *α* = 0.05 and the permutation significance *α* = 0.05. The *p*-values across language ROIs and motor ROIs were corrected for multiple comparison using Benjamini-Hochberg false discovery rate (FDR; Benjamini & Hochberg, 1995).

## RESULTS

### Behavioral Results

We estimated the behavioral competence in L1 and L2 by analyzing the behavioral performance in the semantic judgment task. There was no significant difference between the L1 and L2 in the reaction time (*p* > 0.05, L1 (mean, *SD*): 587.12 ms ± 62.14, L2: 626.20 ms ± 71.59), but the accuracy rate was higher in L1 (96.25% ± 1.8%) than in L2 (91.94% ± 3.9%) (*p* < 0.001).

### General Pattern and Time Course of Activation

The rough level activation timing (grand-averaged sensor waveform across the 204 gradiometers) and spatial distribution (source activation within the major activation peaks) across conditions in the L1 and L2 experiments are shown in Figure 2A and 2B. The source activation map revealed robust activation in the occipital lobe at 130 ms for both the L1 and L2, with slightly greater amplitude for the L1 than the L2. At around 260 ms, activation was found in the posterior temporal area for the L1 and in the lateral occipital-temporal area for the L2. At the peak around 400 ms, a notably greater amplitude was observed for the L1 than the L2. Activation in L1 was broadly distributed to the insular area (partially overlapping with the inferior frontal gyrus), posterior temporal area, anterior temporal area, inferior part of precentral area and central area. For the L2 (mainly the metaphorical condition), robust activation was observed mainly in the posterior temporal area. At around 700 ms, the pattern between L1 and L2 was reversed: L2 showed greater amplitude than L1 in the central and precentral areas.

### Statistical Results

**Figure 4.**
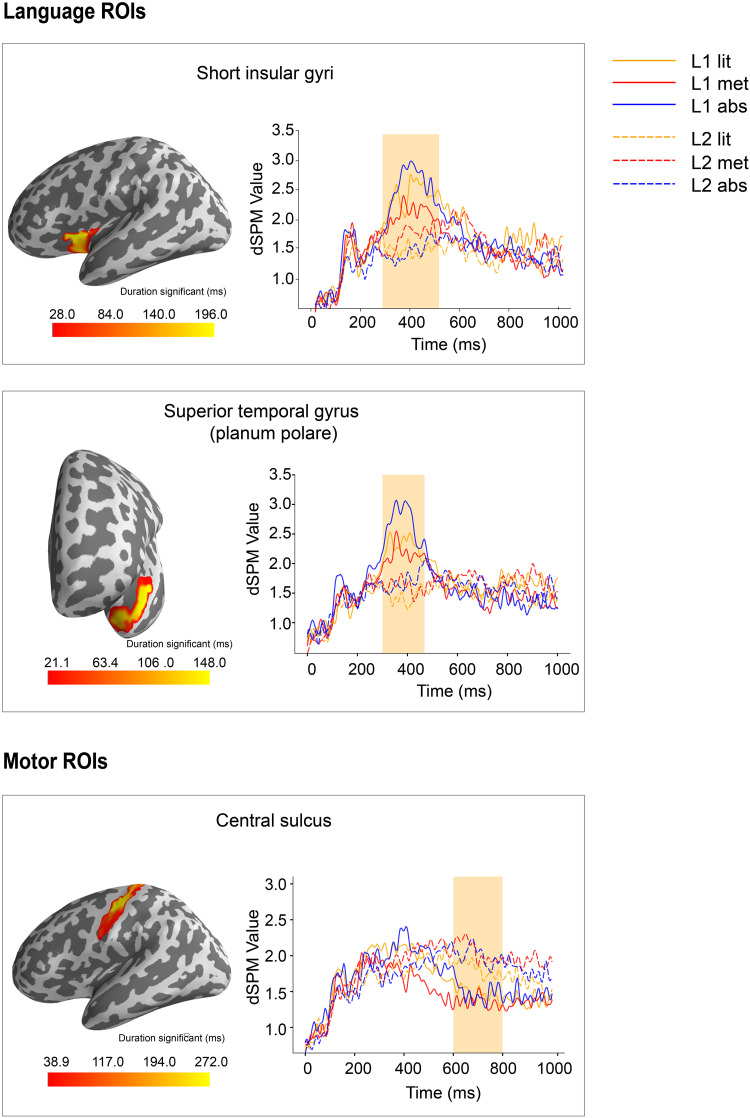
Results of permutation *F* test on the language and motor ROIs. (Left) Significant clusters at source space (clusters exceeding the randomization distribution under H0 hypothesis). The intensity of the color in the cortical map indicates the duration of the time window of clusters. (Right) Source time courses (represented as dSPM value) extracted from significant spatiotemporal clusters; light orange shading area shows time window of the significant cluster. dSPM: dynamic statistical parametric mapping.

Cluster-based permutation *F* test on source data was performed for each language and motor ROI in the TW1 (300–500 ms) and TW2 (600–800 ms) respectively. Statistical results are shown in Table 2. Language and motor ROIs with significant spatiotemporal clusters are shown in Figure 4.

**Table 2.** Statistical results of region-of-interest (ROI) analyses on source data

	Parcellation (Destrieux Atlas a2009s)^a^	Terminologia Anatomica^b^	#Vertice	*p*-values (FDR-corrected)					
				300–500 ms			600–800 ms		
				A:B	A	B	A:B	A	B
Language ROIs	G_insular_short	Short insular gyri	732	0.095	0.042*	0.258	0.243	0.970	0.240
	G_temp_sup-Plan_polar	Planum polare of the superior temporal gyrus	876	0.215	0.042*	0.258	0.406	0.613	0.290
	S_temporal_sup	Superior temporal sulcus	5216	0.095	0.326	0.271	0.298	0.842	0.451
Motor ROIs	S_precentral-inf-part	Inferior part of the precentral sulcus	1587	0.056	0.466	0.859	0.465	0.056	0.246
	S_central	Central sulcus	3139	0.073	0.515	0.512	0.465	0.020*	0.246

*Note*. A:B: interactions between language proficiency and linguistic abstractness; A: the main effect of language proficiency; B: the main effect of linguistic abstractness. The anatomical parcellation was based on Destrieux Atlas a2009s. Statistical significance (*p* < 0.05) is marked in bold with an asterisk.

^a^ Destrieux et al. (2010).

^b^ FIPAT (2019).

For the language ROIs, in the TW1, the cluster-based permutation *F* test revealed a significant main effect of language proficiency in the short insular gyri (*p* = 0.042) and the planum polare of the superior temporal gyrus (*p* = 0.042), manifested as greater activation within these areas for the L1 than for the L2. Statistical analysis did not reveal any significant interaction effect or the main effect of abstractness. In the TW2, no significant effect was found for language ROIs.

For the motor ROIs, no significant effect was found in the TW1. In the TW2, results showed a significant main effect of language proficiency in the central sulcus (*p* = 0.020), manifested as greater activation in the L2 than in the L1. No significant interaction effect or the main effect of abstractness was found.

## DISCUSSION

In this MEG study, we investigated the degree of involvement of the language and motor areas in a language comprehension task. We employed spatiotemporally sensitive MEG recordings, which allowed us to examine the temporal trajectory of language and motor cortex activation. Specifically, we investigated whether the degree of involvement of language and motor areas in the stage of semantic processing was modulated by learner-specific factors (i.e., language proficiency), and/or by stimulus-specific factors (i.e., level of abstractness of the language stimuli).

Our source analysis evidenced a typical spatiotemporal trajectory of visual word processing, which witnessed an early robust activation in the occipital area, followed by activation flowing from the posterior to the anterior temporal and frontal areas (Brennan & Pylkkänen, 2012; Carreiras et al., 2013). In addition, the source estimation results showed neural activation of motor areas across all conditions (literal, metaphorical, and abstract) in both native language (L1) and second language (L2). More importantly, our results showed an overall greater involvement of language areas (short insular gyri and planum polare of the superior temporal gyrus) in the L1 than in the L2 in the time window of 300–500 ms, which has been broadly associated with semantic analysis (Kutas & Federmeier, 2011; Lau et al., 2008; Lau et al., 2013). Although greater activation in the posterior superior temporal sulcus can be seen for the L1 than the L2 in the grand-averaged source results (Figure 2B), it failed to show any statistically significant difference. In addition, our results showed an overall greater involvement of motor area (central sulcus) in the L2 than in the L1 in the late time window of 600–800 ms, which might be associated with post-semantic analysis and integration.

### Compensatory Role of the Motor Cortex in Late-Acquired L2 Processing

Our findings corroborate previous studies in showing that the motor cortex is involved in the processing of not only the L1 but also the L2 (Birba et al., 2020; De Grauwe et al., 2014; Monaco et al., 2021; Tian et al., 2020; Vukovic & Shtyrov, 2014; Zhang et al., 2020). In fact, our results suggest a stronger role for motor areas in the L2 than the L1. Our findings are also in line with earlier studies which suggested that the motor (or sensorimotor) area is involved in the processing of not only action-related but also abstract meaning (Dreyer & Pulvermüller, 2018; Guan et al., 2013; Tian et al., 2020; Vukovic et al., 2017). These findings jointly indicate that motor cortex involvement is ubiquitous in semantic processing, regardless of the linguistic features of the stimuli.

The stronger involvement of the motor cortex in the L2 semantic processing, independent of its linguistic abstractness, allows us to speculate on its role in language processing more generally. The finding is in line with some previous studies showing greater motor activation in the L2 than the L1 (Monaco et al., 2021; Tian et al., 2020), though not exactly in the same time window (275 ms after onset in Monaco et al.’s study, 600 ms in the present study). The somewhat earlier emergence of the effect in Monaco et al. may arise from the use of single verbs, while in our study the stimuli were verb phrases, which are relatively more complex semantically, and may evoke longer-lasting cortical engagement. In addition, the semantic task in Monaco et al.’s study required explicit motor simulation, as participants needed to judge if the verb represents a physical or mental action. In contrast, the task in our study only required the evaluation of semantic congruency and did not require any action-related judgment. Although the underlying process in L1 and L2 may be different between Monaco et al.’s study and ours, both studies indicate stronger involvement of motor areas in L2.

However, there are also contradictory findings. The results of Vukovic and Shtyrov’s study (2014) pointed to greater involvement of the motor cortex in the L1 than the L2, indicated by stronger mu rhythm ERD. This apparently opposite pattern may at least partly be due to the differences in the brain activation measures. ERD (and event-related synchronization) reflects the temporal changes in the power of oscillations, and particularly the 10–20 Hz (hence mu rhythm) is often associated with the level of top-down inhibitory control. Unlike ERD, evoked responses, on the other hand, are time and phase locked to the onset of incoming sensory input and are likely to reflect a different source of neuronal activation. Particularly for the later stages of activation, evoked responses are likely to represent activation of a distributed network, the center of which is represented by the spatial extent of the source model. In their study, Vukovic and Shtyrov interpreted the stronger modulation for the L1 as the results of a more integrated perception-action circuit for the L1 lexical-semantic representation and a higher degree of embodiment for the L1. An alternative interpretation of their findings may, however, be that even though the task did not require verbal output, L1 more readily and automatically engages articulatory preparation, which may manifest as stronger predictive (i.e., top-down) allocation of resources in the motor areas. This interpretation would be in line with the results of anticipatory alpha modulation in visual and language domains (Wang et al., 2018) and challenges the embodied interpretation of the findings. The stronger and automatic recruitment of motor representations in the L1 in early time windows would also be compatible with increased engagement of motor areas in the L2 in later time windows (as shown in our study). Indeed, given the strongly time-evolving nature of language processing in the brain, it is conceivable that the role of the motor cortex may vary across time. As the source result shows in our study, the activation in the L1 (but not the L2) extended to the precentral sulcus in 300–500 ms, although the difference between L1 and L2 did not show statistically significant clusters.

The discussion of the role of the motor cortex in language comprehension may thus need to be approached with increased resolution (both temporally and spatially), as different neuroimaging modalities, and even different neural measures derived by same modality suggest divergent roles. It is also of crucial importance to acknowledge the time-varying nature of language processing. In addition to the methodological concerns, the search for functional significance of motor cortex also requires rigorous use of reasoning in interpreting the neuroscientific findings. Indeed, it needs to be noted that the greater degree of motor cortex activation may not necessarily imply a higher degree of embodiment. As has been pointed out, the involvement of a certain cognitive process cannot be unequivocally inferred from the presence of brain activation of a certain region (cf. reverse inference, e.g., Henson, 2006; Mahon & Hickok, 2016; Poldrack, 2006), as a particular brain region may carry multiple cognitive functions with a primary or secondary role.

The difficulty in specifying the correspondence relationship between brain regions and cognitive functions also applies to neuroimaging studies concerning action-related language processing. Neural activation of the motor cortex has mostly been elucidated as the result of utilizing the motor cortex for mentally simulating action-related meaning. The inference is made based on the established fact that the motor cortex is engaged in motor execution, motor planning, and motor imagery, as has been widely reported (Filimon et al., 2007; Hanakawa et al., 2008; Leonardo et al., 1995). Consequently, motor activations in the studies of semantic processing are believed to indicate the engagement of the motor cortex in the mental simulation of action-related meanings. However, the motor cortex, in addition to its motor-related cognitive functions, has also been shown to be functionally involved in other cognitive processes in a sub-dominant way, including (procedural) memory retrieval, cognitive control, inhibition, and integration (Francis, 2005; Miller, 2000; Mofrad et al., 2020; Lambon Ralph et al., 2017; Ullman, 2004; Willems et al., 2010). In the context of language processing, as mentioned in Maieron et al.’s (2013) study, the engagement of the primary motor cortex may be related to other aspects of cognitive processing rather than specific linguistic processing, which was inferred based on the lack of modulation of language-motor coupling during the action-verb generation task for both lesion and healthy groups.

In the present study, greater activation of the motor cortex was found for the L2 than the L1 across conditions. Referring to the above reasoning, we are of the opinion that the greater activation of the motor cortex may not imply a greater degree of embodiment in the semantic processing of the L2, but a higher demand for cognitive resources to compensate for its lower proficiency and weaker semantic representation compared with the L1. The interpretation is made based on the joint findings of the underactivation of the language areas (short insular gyri and planum polare of the superior temporal gyrus) at the semantic processing stage and the overactivation of the motor area (central sulcus) at the post-semantic processing stage in L2, compared with L1. The planum polare of the superior temporal gyrus, as part of the anterior temporal lobe, has been shown to be a semantic hub for integrating domain-specific concepts and semantic integration in general (Lambon Ralph et al., 2017; see review by Visser et al., 2010). Interpreted in the context of the present study, the L1 with richer semantic representation (compared with the L2) is likely to engage a greater degree of the anterior temporal lobe for meaning processing. In contrast, the weaker semantic representation of L2 may cost longer time for participants to access the meaning of L2, which might account for the early underactivation in language areas. The motor cortex was over-recruited, presumably, to offset the inadequate engagement of language areas, as a result of weaker semantic representation of L2, compared with L1. However, it is important to collect more direct evidence on the causal role of language and motor areas in linguistic tasks, as neuroimaging studies are necessarily correlative in nature.

Similar interpretation about the compensatory mechanism has also been reported in a study of individuals with dyslexia (Richlan et al., 2011), where underactivation in the left temporal region and overactivation in the motor cortex was found in adults with reading difficulty. This lends support to the idea that motor areas may represent general supportive functions in case of lower proficiency. Indeed, it has been validated by converging empirical evidence, that the retrieval of weakly encoded information relies more strongly on the control network (Lambon Ralph et al., 2017). Based on the above discussion, we argue that the greater activation of the motor cortex in the L2 may not signify a higher degree of embodiment, but a higher demand for cognitive resources to compensate for the inadequate engagement of the language network.

### Functional Role of the Motor Cortex

By clarifying the role of learner-specific (i.e., language proficiency) and stimulus-specific (i.e., abstractness) factors, our findings shed light on the functional role of the motor cortex in language processing. There has been a longstanding debate on the functional and epiphenomenal role of motor cortex involvement in the literature on embodied language processing (Bocanegra et al., 2017; Desai et al., 2015; Fernandino et al., 2013; García et al., 2019; Reilly et al., 2019; Repetto et al., 2013; van Elk et al., 2010; Vukovic et al., 2017). Similar to our paradigm, some earlier studies attempted to disentangle these two roles by referring to the time course of the motor cortex activation, compared with that of the language areas (García et al., 2019; Papeo et al., 2009; Reilly et al., 2019; van Elk et al., 2010). The motor-related activations or modulations occurring at an early stage of semantic processing (130–190 ms post-stimulus in García et al., 2019; 300 ms in Reilly et al., 2019; 400 ms in van Elk et al., 2010) are considered as evidence supporting the assumption of the functional (i.e., necessary) role, which claims that the motor cortex directly contributes to semantic processing, while activations occurring at a later stage are considered to reflect post-semantic motor imagery (500 ms in Papeo et al., 2009) and not necessarily contributing to language comprehension.

However, the onset of semantic processing is unlikely to be clearly defined by a fixed time point, and it may vary considerably depending on learner-related factors (e.g., language proficiency and language experience) and language-related factors (e.g., language distance). For a less proficient language, the latency of lexical-semantic retrieval and integration can be delayed compared with the highly proficient native language. Considering the influence of language proficiency, we assume that the greater activation of the motor cortex in the L2 in our study is not the result of post-semantic motor imagery but reflects the general cognitive processes that support semantic processing in an indirect way. It may thus be useful for the discussion of the functional or epiphenomenal role of the motor network to focus not only on latency of motor cortex activation, but also on language proficiency, which may lead to variance in the latency of semantic access.

### The Null Effect of Abstractness

Our study did not reveal any significant effect of abstractness, suggesting that neural responses in the motor areas may not be modulated by the degree of abstractness of the linguistic input. The finding is inconsistent with our prediction of decreased motor involvement with the increase of abstractness. Our finding is also inconsistent with previous studies exploring the effect of abstractness on a continuum (i.e., literal, metaphorical (idiomatic), and abstract; Desai et al., 2013; Tian et al., 2020). In their studies, hierarchically attenuated motor activation was found with the increase of linguistic abstractness.

So far, most studies concerning the effect of abstractness on motor cortex involvement mainly focused on literal and figurative action-related language (mainly metaphorical and idiomatic). Some revealed greater involvement of motor cortex for literal than figurative language (Cacciari et al., 2011), and some reported a similar degree of motor cortex involvement between them (Boulenger et al., 2009; Boulenger et al., 2012). Inconsistently, some other studies found motor cortex involvement only for the literal language, but not for the figurative (Raposo et al., 2009). The discrepancy in findings may derive from methodological differences across studies, including task demands (covert vs. overt motor association), stimulus properties (word vs. phrase vs. sentence), and ways of presentation (word-by-word vs. whole item, visually vs. aurally). As has been highlighted, the recruitment of the motor cortex in action semantic processing is task (Giacobbe et al., 2022; Tomasino et al., 2008) and context dependent (Raposo et al., 2009).

Moreover, current findings call for a reflection on the relationship between artificial categorization of abstractness and its actual brain response. Although the stimuli do follow a linguistically defined continuum of abstractness, the actual brain responses may not follow such gradation. In future studies, it will be important to test the modulatory effect of abstractness on the degree of motor cortex recruitment by using comparable approaches.

### Limitations

Our study has some limitations. First, our study is a correlative study in nature, and interpretations are mainly “bound” to earlier literature. Second, our study only included ROI-based analysis motivated by its hypothesis-driven nature. The exclusion of whole-brain analysis may cause the ignorance of important neural activity in other brain regions. Future studies should further investigate the relationship between language and motor networks in bilingual language processing by employing comparable approaches.

### Conclusion

Our study explored the degree of involvement of language and motor areas modulated by language proficiency and linguistic abstractness. We reported an overall greater activation in the language areas for the L1 than the L2 at the semantic processing stage at 300–500 ms, and an overall greater activation in the motor regions for the L2 than the L1 at the later post-semantic processing stage at 600–800 ms. The over-recruitment of the motor areas in the L2 implied a compensatory role of the motor area to offset the lower language proficiency of the L2 in relative to the L1. Our study provides an alternative interpretation of motor cortex involvement in language processing and invites further research to explore the factors that modulate this relationship.

## ACKNOWLEDGMENTS

The authors would like to thank Aino Sorsa for her assistance in data collection and Dr. Viki-Veikko Elomaa for program setup. The authors would also like to thank Dr. Weiyong Xu, Dr. Xiulin Wang, Dr. Simo Monto, and Erkka Heinilä for their assistance and suggestions in data analysis.

## FUNDING INFORMATION

Lili Tian, China Scholarship Council (https://dx.doi.org/10.13039/501100004543), Award ID: 201708500099.

## AUTHOR CONTRIBUTIONS

**Lili Tian**: Conceptualization; Formal analysis; Investigation; Writing – original draft; Writing – review & editing. **Hongjun Chen**: Conceptualization. **Pyry Petteri Heikkinen**: Formal analysis. **Wenya Liu**: Formal analysis. **Tiina Parviainen**: Conceptualization; Resources; Writing – review & editing.

## DATA AND CODE AVAILABILITY STATEMENTS

The data are not publicly available due to the restrictions of research ethics stated in the Privacy Notice for Research Subjects in terms of the privacy of research participants.

The data that support the findings of this study are available upon reasonable request from Tiina Parviainen (tiina.m.parviainen@jyu.fi) and Lili Tian (litian@jyu.fi).

In compliance with the General Data Protection Regulation, the following situation will be approved when requesting the data: (1) actions aiming to confirm and verify the validity and authenticity of the results of the current research; (2) actions related to scientific research or other compatible purpose.
